# Numerical Material Testing of Mineral-Impregnated Carbon Fiber Reinforcement for Concrete

**DOI:** 10.3390/ma17030737

**Published:** 2024-02-03

**Authors:** Kai Zernsdorf, Viktor Mechtcherine, Manfred Curbach, Thomas Bösche

**Affiliations:** 1 Department of Structural Engineering, Hochschule für Technik und Wirtschaft Dresden, 01069 Dresden, Germany; thomas.boesche@htw-dresden.de; 2 Institute of Construction Materials, Technische Universität Dresden, 01069 Dresden, Germany; viktor.mechtcherine@tu-dresden.de; 3 Institute of Concrete Structures, Technische Universität Dresden, 01069 Dresden, Germany; manfred.curbach@tu-dresden.de

**Keywords:** mineral-impregnated carbon fiber, damage mechanics, representative volume elements, numerical material testing

## Abstract

This work was dedicated to the simulation of fiber composite structures consisting of carbon fibers and mineral impregnation. The aim of this study was to generate a micromodel that predicts the properties of a mineral-impregnated carbon fiber reinforcement. The numerical characterization was based on the discrete microscopic modeling of the individual phases using a representative volume element. In addition, the stochastic nature of the fiber strength, the anisotropic damage mechanisms of the brittle matrix, and the non-linear bonding behavior between the filaments and the matrix were considered in the material models. The material models were adjusted based on the literature sources and our own experimental investigations. This was followed by the validation of the representative volume element, quantifying the evolution of stiffness and damage under longitudinal tensile loading. The numerical results of material stiffness, as well as the tensile strength of the representative volume element, were compared with the results of the experimental investigations. To verify the robustness of the numerical model, significant model parameters were subjected to a sensitivity analysis.

## 1. Introduction

The development of mineral-impregnated carbon fiber reinforcement (MCF reinforcement), which is used as a tension member in concrete, is an innovative approach that goes hand in hand with the substitution of temperature-sensitive organic impregnation matrices. These efforts were initiated with the development of mineral impregnation matrices suitable for high-filament-count carbon rovings [1,2,3]. Initial investigations into the bonding behavior of MCF reinforcement with the surrounding concrete and experimental investigations into the longitudinal stiffness and strength have already been carried out [1,3]. Given their exceptional corrosion resistance, superior bonding characteristics, and excellent heat resistance, concrete structures reinforced with MCF offer a diverse range of applications in the field of structural engineering [1,2,3]. However, the applicability of MCF reinforcement in concrete requires detailed knowledge of the mechanical performance of the reinforcement.

An excellent method for assessing the performance of a novel composite, as well as the individual phases in the composite, is numerical modeling of the continuum at the microscopic level. This method demonstrates the optimization potential of the novel composite without the need for many trials. A numerical simulation also provides a detailed understanding of the damage mechanisms, which, in turn, allows a better understanding of the MCF reinforcement. Moreover, it is possible to predict the macroscopic material response by homogenizing the microscopic model. Such homogenization can be achieved via computer-generated representative volume elements (RVEs). The homogenization of an inhomogeneous, microscopic structure is only feasible if the following conditions are satisfied [4]:

1. The principle of scale separation is observed;
2. An averaging theorem is implemented;
3. The Hill condition [5] continues;
4. The constitutive behavior of the individual phases in the RVE can be described;
5. A representative volume element (RVE) exists.

Regarding 1, according to the principle of scale separation, the RVE must be of sufficient size to capture all microstructural information, while, at the same time, being significantly smaller than the macroscopic structure. In this case, the linkage of any material point at the macrolevel with the RVE will be possible, and macroscopic stresses and strains will occur uniformly in the microstructure [4].

Regarding 2, the conditions of the averaging theorem are satisfied if the strain or stress at any point in the macromodel equals the volume average of the strains or stresses in the micromodel [4].

Regarding 3, the Hill condition [5] states that the stress at a macroscopic material point is equal to the volume average of the stresses of the associated micromodel.

Regarding 4, the behavior of the individual phases in the RVE must be identified before the simulation. Phenomenological material models must be available for the individual phases of the RVE [4]. The variables of the material models must be determined experimentally, analytically, and/or inversely.

Regarding 5, using homogenization methods, the macroscopic response corresponds to the mean value of the microscopic responses of a sample that is defined to a finite size. The defined microscopic sub-area must represent the entire microstructure on average. The individual phases of the microstructure must be in such a form that a representative section within the microstructure can be obtained. If this is the case, a representative volume element (RVE) exists [4].

**Figure 1:**
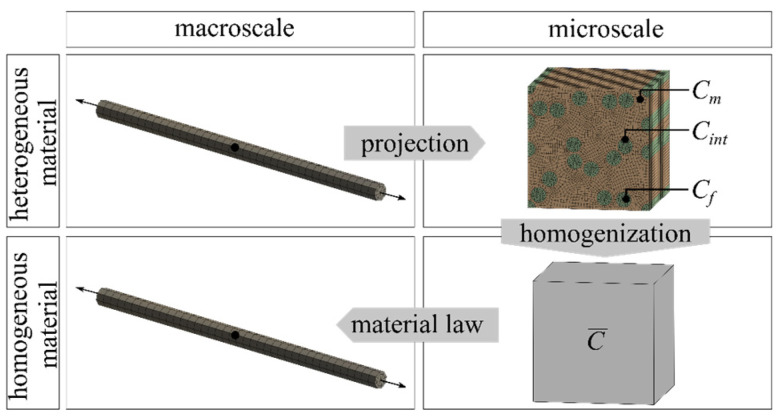
Decoupled multiscale analysis [4]. © Kai Zernsdorf.

There are two different approaches to computer-assisted homogenization in the literature. The continuous computational homogenization approach (also called the FE² approach) can be used, in which the microscopic boundary-value problem is coupled with the deformation of the corresponding RVE at a given macroscopic material point and solved via a nested finite element analysis (amongst others [6,7]). Alternatively, the decoupled method can be used, which is presented in [8] and is outlined in Figure 1. In this case, one or more RVEs are analyzed in advance using numerical simulations. Subsequently, the macroscopic stress response is determined via the homogenization of the RVE and based on this, a suitable macroscopic material law is defined. The application of decoupled multiscale analysis to carbon fibers with a plastic impregnation is presented in [9]. This method provides insight into the dominant effects at the microlevel and enables sensitivity studies of the material parameters and structural parameters. This method has been applied to the study of concrete, among other areas, as reported in [10], and was applied in this work.

A significant factor that influences the response of an RVE is the choice of boundary conditions. The chosen boundary conditions are applied to the boundaries of the RVE and simulate the surrounding medium [4]. 

Studies by Böhm [11] have shown that periodic boundary conditions achieve the most valid results within a numerical simulation. In accordance with this observation, this study also applied periodic boundaries. The theoretical aspects of this type of boundary conditions are explained in more detail in [4,11]. An explanation of the implementation of the boundary conditions in the numerical model is provided in Section 2.2. The approach suggested in [12] corresponds the most to the parameters chosen for this study. In [12], a micromechanical model predicts the stiffness degradation of the composite material. The model considers the random filament distribution, the fiber strength, a non-linear bond model, and the damage behavior of a polymeric matrix.

Deviating from previous studies, in the present work, we developed an RVE made of carbon filaments with a mineral impregnation matrix (MCF-RVE) (Section 2). The scale transition was in the form of a decoupled homogenization approach. The RVE considered both the random filament distribution and the non-linear material behavior of the individual components, as well as their bonding interactions, which are presented in Section 3. The variables of the material models of the individual phases in the MCF-RVE are adjusted in Section 4 based on the literature sources and experimental investigations. The validation of the calibrated MCF-RVE is presented in Section 5 and was conducted via a comparison between the homogenized macroscopic stress–strain response of the MCF-RVE and the experimental uniaxial tensile tests carried out by Wilhelm [1]. By using the validated MCF-RVE, it was also possible to realistically represent the MCF reinforcement at the component level using appropriate material models. As a result, deformations, crack formations, and the maximum load-bearing capacities of the composite material could be predicted numerically and analytically, without having to carry out an extensive series of experimental investigations in advance. All simulations were conducted on a desktop computer equipped with an AMD Ryzen 9 5950X 16-core processor from Advanced Micro Devices, Inc. (Santa Clara, CA, USA) and 128 GB of RAM from Micron Technology, Inc. (Boise, ID, USA).

## 2. FEM

The numerical simulation was carried out using the FE program Ansys (version 21.1). This enabled a realistic simulation of the linear and non-linear material behaviors of the carbon filaments and the mineral phase. Furthermore, it facilitated the non-linear simulation of the bonding interactions between both components.

**Figure 2:**
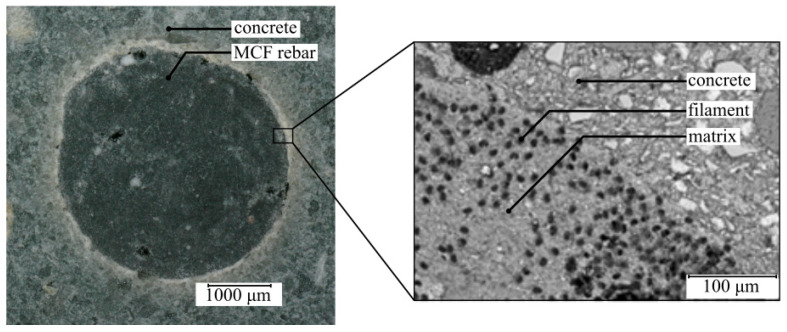
ESEM images of cross-sections of MCF reinforcement in concrete. Source: [3]. © Kai Zernsdorf.

First, the construction of a numerical geometric model is detailed in this section, which represents the structure of the MCF reinforcement at the microscopic level. The reinforcement consists of uniaxial aligned carbon filaments embedded in a mineral matrix. The fiber volume fraction φ is an essential factor influencing the geometry and mechanical properties of the MCF-RVE. According to Wilhelm [1], the average fiber volume fraction of MCF reinforcement (Figure 2) is 17%.

### 2.1. Model Geometry

Ensuring an average representation of all relevant geometric and mechanical properties of the MCF reinforcement in the RVE requires the following:

1. A sufficient quantity of heterogeneities that are randomly distributed. In this case, heterogeneity refers to the filaments embedded in the mineral phase of the MCF-RVE.
2. Geometric periodicity.
3. The exclusion of discontinuities in the deformation field.

The numerical micromodel’s side length $L_{RVE}$ corresponds to an integer multiple of the diameter of a filament $d_{f}$. The carbon filament modeled in this study had a diameter of 6.9 µm, according to [1]. Five distinct side lengths—namely, $L_{RVE}=3\times 3$, $L_{RVE}=5\times 5$, $L_{RVE}=8\times 8$, $L_{RVE}=10\times 10$, and $L_{RVE}=12\times 12$ (shown in Figure S1)—were analyzed to determine the optimal dimension of the representative volume element. The evaluation of the investigations is provided in Figure S2.

The randomized arrangement of circular filaments within the model geometry was generated using an algorithm external to the program. The programmed algorithm for the random distribution of the filaments in the MCF-RVE was based on a hard-core model (also called a random sequential adsorption model), which was implemented using the program MATLAB (version 9.12). A flowchart of the algorithm is provided in Figure S3. Within this model, a random collection of non-overlapping filaments was generated (cf. [13,14]). The filaments, with diameter $d_{f}$, were positioned one after the other over their center points. Positioning was even over the region of the MCF-RVE. If a new filament did not overlap with one of the filaments already created, its position was fixed. Otherwise, the positioning of the filament was discarded. To ensure geometric periodicity, the surfaces of the filaments that penetrated the edge of the MCF-RVE were mirror-shifted to the opposite side surfaces. Thus, opposite boundary nodes coincided and formed the periodic boundary of the model. The algorithm was completed when the fiber volume fraction φ of the MCF-RVE was equal to the target value, which was 17% in the context of this work. A flowchart of the algorithm for the random filament arrangement using the HCM is shown in [15,16,17].

**Figure 3:**
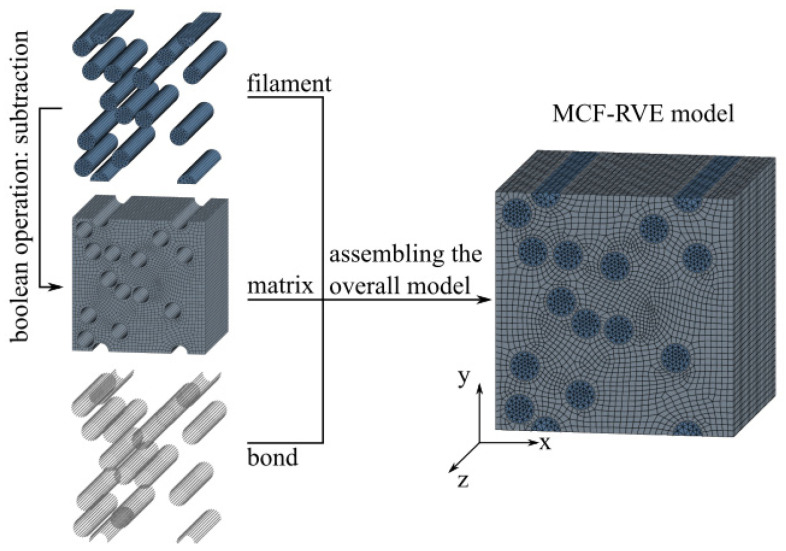
Example geometric model of an MCF-RVE. © Kai Zernsdorf.

The remaining area of the MCF-RVE was filled with a mineral impregnation matrix. In the context of this work, the matrix corresponded to a homogeneous, undisturbed continuum. Finally, spring elements were created between the filaments and the mineral matrix, representing the bonding interaction between the phases. Additional numerical models that were generated are depicted in Figure 3, Figures S1 and S5.

### 2.2. Boundary Conditions

**Figure 4:**
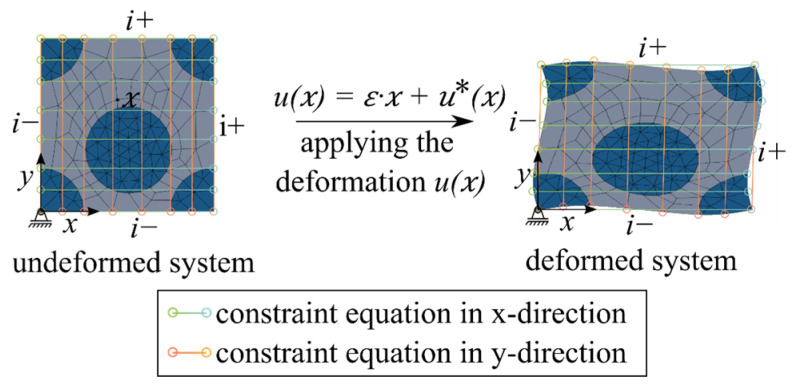
Application of periodic boundary conditions. © Kai Zernsdorf.

To avoid discontinuities in the deformation field, the RVE was coupled with periodic boundary conditions in addition to geometric periodicity. These caused the stress–strain fields in the RVE on opposite sides to remain periodic [18]. The periodic boundary conditions are illustrated in an example of a two-dimensional RVE (Figure 4). A periodic deformation boundary condition that is transverse to the filament $u\left(x\right)$ (cf. Figure 3) for a continuum with heterogeneity leads to a linearly distributed displacement field $\varepsilon^{0}\cdot x$ and additional fluctuations $u^{*}\left(x\right)$ [19]:

(1) $$u\left(x\right)=\varepsilon^{0}\cdot x+u^{*}\left(x\right)$$

where $\varepsilon^{0}$ corresponds to the strain at the macroscopic level.

For each RVE, the opposite edge nodes must be divided into pairs. The node displacements of the edge node pairs are as follows (cf. [19]):

(2) $$u^{i+}=\varepsilon^{0}\cdot x^{i+}+u^{*}\left(x\right)\;u^{i-}=\varepsilon^{0}\cdot x^{i-}+u^{*}\left(x\right)$$

where the indices i+ and i− indicate the *i*th pair of two opposite edge nodes of the RVE. The fluctuation $u^{*}\left(x\right)$ is the same for the node pairs due to the periodicity of the deformation boundary conditions (Figure 4). Therefore, the difference in node displacements results in

(3) $$u^{i+}-u^{i-}=\varepsilon^{0}\cdot \left(x^{i+}-x^{i-}\right)=\varepsilon^{0}\cdot \Delta x^{i}$$

The distance between two adjacent node pairs $\Delta x^{i}$ and $\varepsilon^{0}$ is constant for each node pair. Therefore, the expression shown in Equation (3) could be implemented in the FE model via node-by-node constraint equations.

The implementation of periodic boundary conditions in a numerical model is explained in greater detail in [20].

### 2.3. Homogenization

The response of an MCF-RVE under different loading conditions corresponds to the homogenized stress $\bar{\sigma }$ of the model. Based on the local stress fields $\sigma^{loc}$ of the MCF-RVE, the homogenized stress at each time step can be calculated using the following equation:

(4) $$\bar{\sigma }=\frac{1}{V}\int_{V}^{\;}\left(\sigma^{loc}\right)dV$$

where V represents the volume of the MCF-RVE. It is worth noting that non-linear effects within the MCF-RVE, such as bond or filament degradation, are accounted for in the homogenized stress.

## 3. Material Models of the Individual Phases

The geometric model of the MCF-RVE has three phases with three different material models (filaments ${\left(∎\right)}_{f}$, mineral matrix ${\left(∎\right)}_{m}$, and the bonds between the filaments and the matrix ${\left(∎\right)}_{int}$). To accurately depict the mechanical effects, it is imperative to employ appropriate material models that define the pertinent characteristics of the distinct phases and their interrelationships. In the following sections, the most important features of the constitutive models used are described and explained in mathematical form. Furthermore, the implementation of the material models in the numerical model is described.

### 3.1. Carbon Filament

Carbon filaments exhibit linear elastic material behavior up to their failure and transversely isotropic material properties [18,19]. The stress–strain behavior of each filament element can thus be formulated using Hooke’s law:

(5) $$\sigma_{f}=C_{f}:\varepsilon_{f}$$

For transversely isotropic materials, the stiffness tensor $C_{f}$ can be expressed in Voigt notation as follows:

(6) $$C_{f}={\left[\begin{array}{cccccc} \frac{1}{E_{f,x}} & -\frac{\upsilon_{f,yx}}{E_{f,y}} & -\frac{\upsilon_{f,zx}}{E_{f,z}} & 0 & 0 & 0\\ -\frac{\upsilon_{f,xy}}{E_{f,x}} & \frac{1}{E_{f,y}} & -\frac{\upsilon_{f,zy}}{E_{f,z}} & 0 & 0 & 0\\ -\frac{\upsilon_{f,xz}}{E_{f,x}} & -\frac{\upsilon_{f,yz}}{E_{f,y}} & \frac{1}{E_{f,z}} & 0 & 0 & 0\\ 0 & 0 & 0 & \frac{1}{G_{f,yz}} & 0 & 0\\ 0 & 0 & 0 & 0 & \frac{1}{G_{f,xz}} & 0\\ 0 & 0 & 0 & 0 & 0 & \frac{1}{G_{f,xy}} \end{array}\right]}^{-1}$$

where $E_{f}$ denotes the modulus of elasticity, $\nu_{f}$ is Poisson’s ratio, and $G_{f}$ indicates the shear modulus. The indices x, y, and z refer to the three spatial directions of the filament. It should be noted that the longitudinal direction of the filament according to Equation (6) corresponds to the third spatial direction (Figure 3). The elastic parameters can be found in the literature, e.g., [21].

To describe the damage behavior of the filament elements, a one-dimensional isotropic damage law was applied, which is described in [12,22]. In this study, the damage threshold value of a filament was defined according to Tavares et al. [12], using the randomized tensile strength of each filament element $X_{f,z}$ based on a Weibull distribution [23] with the following equation:

(7) Xf,z=σ0−L0Lln1−Xz1m

where $\sigma_{0}$ describes the characteristic tensile strength of a filament with a gauge length $L_{0}$ in the longitudinal direction of the filament. In the context of this work, the filament length is denoted by L and is equivalent to the length of a filament element in the longitudinal direction. $X_{z}$ represents a random number between 0 and 1 that is assigned to the element. m describes the Weibull module. Each carbon filament exhibits linear elastic material behavior up to the damage threshold value $X_{f,z}$.

The damage law is implemented by reducing the stiffness tensor $C_{f}$ through the degradation $D_{f}\left({\hat{\gamma }}_{t}\right)$:

(8) $$C_{f}^{D}=C_{f}\left(1-D_{f}\left({\hat{\gamma }}_{t}\right)\right)$$

With the introduction of the damaged stiffness tensor $C_{f}^{D}$, Hooke’s law (Equation (5)) can be extended to the variable $D_{f}\left({\hat{\gamma }}_{t}\right)$ as follows:

(9) $$\sigma_{f}=C_{f}\left(1-D_{f}\left({\hat{\gamma }}_{t}\right)\right):\varepsilon_{f}$$

When implementing the formulated material model into the FE model, it is important to ensure that the degree of degradation $D_{f}\left({\hat{\gamma }}_{t}\right)$ does not decrease. It is, therefore, appropriate to define a history variable ${\hat{\gamma }}_{t}$ for each element. This indicates the maximum value of stress reached in the longitudinal direction of the filament in the loading history. Thus, the degree of damage is no longer dependent on the stress of the element but on the history variable, which is defined based on the degree of maximum stress in the longitudinal direction of the filament. If the stress at time point t is less than the history variable at time point t−1, the history variable at time point t is equal to the history variable at time point t−1 [22]:

(10) $${\hat{\gamma }}_{t}=max\left({\hat{\gamma }}_{t-1},\sigma_{f,t}\right)$$

The degradation $D_{f}\left({\hat{\gamma }}_{t}\right)$ of a carbon filament is defined in the form of an interval function:

(11) $$D_{f}\left({\hat{\gamma }}_{t}\right)=\left\{\begin{array}{c} 0\;für\;{\hat{\gamma }}_{t}<X_{f,z}\\ 1\;für\;{\hat{\gamma }}_{t}\geq X_{f,z} \end{array}\right.$$

If the history variable ${\hat{\gamma }}_{t}$ of an element is greater than the randomized tensile strength of the element $X_{f,z}$, the degradation $D_{f}\left({\hat{\gamma }}_{t}\right)$ assumes a value of 1.

The described material model was implemented in the FE simulation using a macro. The overall sequence of the programmed algorithm was adapted and verified according to Barbero [22] and is depicted in Table 1. Each element was degraded using the element death option in the FE program Ansys (version 21.1) [24].

### 3.2. Mineral Matrix

The elastic damage microplane material model was used to describe the material behavior of the mineral phase. This model was taken from [25]. The matrix was assumed to be homogeneous, as already mentioned in Section 2. In the material model, pores or particles were regarded as being smeared. The material model used for this study is available in the FE program and considers multi-axial stress conditions, as well as induced anisotropic material behavior. It should be noted that different microplane models exist with different projection forms of stresses and strains, as well as constitutive laws. An overview of these models is provided by Leukart [25]. In the present work, the model was formulated in a thermodynamically consistent manner. This means that the degree of energy ψ input at the material point ${\left(∎\right)}^{mac}$ was equal to the sum of the energy inputs of the microplanes associated with the material point ${\left(∎\right)}^{mic}$:

(12) $$\psi^{mac}=\frac{3}{4\pi }\int_{\Omega }^{\;}\psi^{mic}d\Omega$$

It was integrated over the angle Ω of a unit sphere with a surface of 4π. In addition, the element distortions were projected onto the individual microplane levels via a volumetric–deviatoric split as follows:

(13) $$\varepsilon_{vol}=vol:\varepsilon \;\varepsilon_{dev}=dev:\varepsilon$$

where $\varepsilon_{vol}$ and $\varepsilon_{dev}$ represent the volumetric and deviatoric portions of the second-order strain tensor [25], respectively; vol corresponds to the volumetric portion of the second-order unit tensor; and dev corresponds to the deviatoric portion of the second-order unit tensor.

The material behavior of the mineral matrix is isotropic in the undamaged state and can be described using Hooke’s law according to Equation (5). Since the stiffness tensor of the mineral matrix $C_{m}$ only has two independent material constants, unlike the transversely isotropic material behavior of the filaments, Equation (5) can be applied to the matrix in the following form:

(14) $$\sigma^{mac}=3K^{mac}\varepsilon_{vol}+2G^{mac}\varepsilon_{dev}$$

where $K^{mac}$ describes the bulk modulus, and $G^{mac}$ describes the shear modulus. 

To enable the induction of damage, Equation (14) is extended by the damage variable $d^{mac}$ as follows:

(15) $$\sigma^{mac}=\left(1-d^{mac}\right)\left(3K^{mac}\varepsilon_{vol}+2G^{mac}\varepsilon_{dev}\right)$$

The ratio between the macroscopic and microscopic bulk modulus and shear modulus is defined as follows:

(16) $$K^{mic}=3\;K^{mac}\;G^{mic}=G^{mac}$$

The stresses on each microplane level result from the derivation of the free Helmholtz energy $\psi^{mic}$, according to the respective strain component:

(17) $$\sigma_{v}^{mic}=\frac{\partial \psi^{mic}}{\partial \varepsilon_{vol}}=K^{mic}\varepsilon_{vol}\;\sigma_{d}^{mic}=\frac{\partial \psi^{mic}}{\partial \varepsilon_{dev}}=2G^{mic}\varepsilon_{dev}$$

Combining Equations (12), (15) and (17) yields the following element stress:

(18) $$\sigma_{m}^{mac}=\frac{3}{4\pi }\int_{\Omega }^{\;}\left(1-d^{mic}\right)\left(K^{mic}V\varepsilon_{vol}+2G^{mic}Dev^{T}\varepsilon_{dev}\right)d\Omega$$

The initiation and progression of damage are defined using the equivalent strain energy $\eta^{mic}$. This results from the first invariant of the strain tensor, the second invariant of the deviatory part of the strain tensor, and the variables $k_{0}$, $k_{1}$, and $k_{2}$:

(19) $$H^{mic}=k_{0}I_{1}+\sqrt{k_{1}^{2}I_{1}^{2}+k_{2}J_{2}}$$

The variables $k_{0}$, $k_{1}$, and $k_{2}$ depend on the variable k that determines the ratio of the uniaxial compressive strength to the tensile strength:

(20) $$k_{0}=k_{1}=\frac{k-1}{2k\left(1-2\nu_{m}\right)}\;k_{2}=\frac{3}{k{\left(1+\nu_{m}\right)}^{2}}$$

where $\nu_{m}$ corresponds to Poisson’s ratio of the matrix.

The evolution of the damage is represented by the following function as a function of $\eta^{mic}$:

(21) $$d^{mic}=1-\frac{\gamma_{0}^{mic}}{\eta^{mic}}\left(1-\alpha^{mic}+\alpha^{mic}\exp\left(\beta^{mic}\left(\gamma_{0}^{mic}-\eta^{mic}\right)\right)\right)$$

In this context, $\alpha^{mic}$ defines the maximum degree of degradation, $\beta^{mic}$ indicates the degradation rate, and $\gamma_{0}^{mic}$ denotes the damage threshold value. It should be noted that the value of the damage $d^{mic}$ cannot decrease over the history of the stress.

The non-linear elastic material model can be implemented with the scalar variables k, $\alpha^{mic}$, $\beta^{mic}$, and $\gamma_{0}^{mic}$ in the numerical model. In addition, the linear elastic isotropic material behavior $\nu_{m}$ is defined based on the modulus of elasticity $E_{m}$ and Poisson’s ratio $\nu_{m}$.

**Figure 5:**
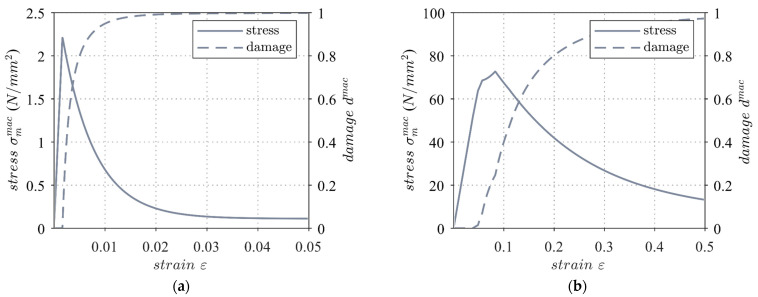
Stress–strain behavior and degradation measure of the elastic damage microplane material model under uniaxial stress (**a**) and compression (**b**). © Kai Zernsdorf.

The stress–strain behavior of the uniaxial tensile strain state for a single volume element, as well as the macroscopic degradation $d^{mac}$ as a function of strain, resulting from the presented material model is shown in Figure 5. The damage at the macroscopic level is defined as follows:

(22) $$d^{mac}=\frac{1}{4\pi }\int_{\Omega }^{\;}d^{mic}d\Omega$$

### 3.3. Bonds between the Filaments and the Matrix

The bonds between the filaments and the matrix has a crucial effect on the mechanical properties of the composite material [12]. In this study, spring elements were used to model the contact between the filaments and the matrix. For this purpose, the spring model of Ngo and Scrodelis [26] was used, which was developed for modeling the bond between a filament and concrete. An adaptation of this model to the composite material considered in this work was carried out. In this adaptation, neighboring node pairs of the filament and matrix elements were coupled via springs.

**Figure 6:**
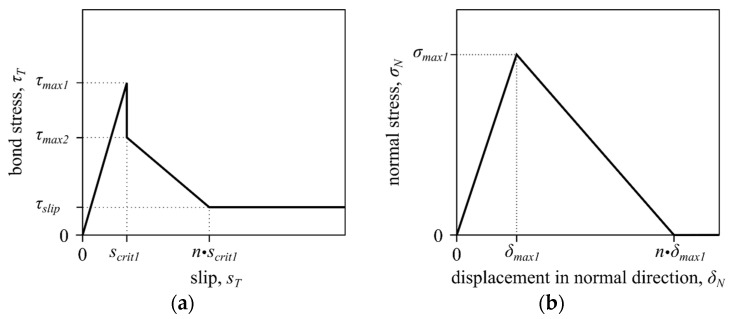
Bond model in the tangential direction [27] (**a**) and normal direction [8,34,35,36] (**b**). © Kai Zernsdorf.

An analytical bond model was used to approximate the mechanical properties of the spring elements. This model enabled the characterization of the bonding behavior, considering the material degradation as a function of the relative displacement [27,28,29,30,31,32]. In this study, two interval functions were used for the analytical description of the bond behavior. For the description of the shear behavior between the carbon filaments and the mineral matrix, Zhandarov and Mäder [33] recommend a multilinear bond model according to Brameshuber and Banholzer [27]. This model is subdivided into three parts and is suitable for describing bond behavior with a peak and a subsequent drop to a constant bond stress level. The following function describes the analytical bond model according to Brameshuber and Banholzer [27] and is shown in Figure 6a:

(23) $$\tau_{T}\left(s_{T}\right)=\left\{\begin{array}{c} \frac{\tau_{max1}}{s_{crit1}}\cdot s_{T}\;\;\;\;\;\;\;\;\;\;\;0<s_{T}<s_{crit1}\\ \tau_{max2}-\frac{\tau_{max2}-\tau_{slip}}{n\cdot s_{crit1}-s_{crit1}}\cdot s_{T}s_{crit1}<s_{T}<n\cdot s_{crit1}\\ \tau_{slip}\;\;\;\;\;\;\;\;\;\;\;\;\;\;\;s_{T}>n\cdot s_{crit1} \end{array}\right.$$

where $\tau_{t}\left(s_{t}\right)$ represents the effective mean bond stress as a function of the slip $s_{t}$; $\tau_{max1}$ denotes the maximum effective bond stress with the associated slip $s_{crit1}$; $\tau_{max2}$ defines the maximum bond stress after exceeding $s_{crit1}$; and the factor n defines the maximum slip during the transition into the constant frictional bond stress $\tau_{slip}$ as a function of $s_{crit1}$.

The bond behavior in the normal direction to the filaments is described with the multilinear normal stress–displacement ratio (Figure 6b). The mechanical behavior of the description of bond behavior in the normal direction has been documented in many research works [8,34,35,36] and is represented as follows:

(24) $$\sigma_{N}\left(\delta_{N}\right)=\left\{\begin{array}{c} \begin{array}{c} \frac{\sigma_{max1}}{\delta_{max1}}\cdot \delta_{N}\;0<\delta_{N}<\delta_{max1}\\ \sigma_{max1}-\frac{\sigma_{max1}}{n\cdot \delta_{max1}-\delta_{max1}}\cdot \delta_{N}\delta_{max1}<\delta_{N}<n\cdot \delta_{max1} \end{array}\\ 0\;\delta_{N}>n\cdot \delta_{max1} \end{array}\right.$$

There are currently no experimental results available to quantify the bond behavior between the filaments and the mineral matrix normal to the filaments. Therefore, the parameters had to be adjusted according to the parameters of the bond stress–displacement ratio [37] as follows:

(25) $$\sigma_{max1}=\frac{\tau_{max1}}{100}$$

(26) $$\delta_{max1}=\frac{s_{crit1}}{100}$$

The laws described in Equations (23) and (24) could be implemented within the numerical model using two-notched, non-linear spring elements (COMBIN39). Spring adjustment was carried out as described in Section 4.3 based on experimental investigations. Unlike the material models of the filaments (Section 3.1) and matrix (Section 3.2), the mechanical properties of the bonding model were not defined using a material model but via real constants assigned to the COMBIN39 elements. 

The connection data for the force–displacement ratio in the tangential direction were determined by converting Equation (23) into a force–slip relationship according to Equation (27):

(27) $$F_{T}\left(s_{T}\right)=\left\{\begin{array}{c} \frac{\frac{\tau_{max3}}{s_{crit2}}\cdot u_{f}l_{v}\cdot s_{T}}{n_{combin}}0<s_{T}<\delta_{max1}\\ \frac{\tau_{max2}\cdot u_{f}l_{v}-\frac{\tau_{max2}-\tau_{slip}}{n\cdot s_{crit1}-s_{crit1}}\cdot u_{f}l_{v}\cdot s_{T}}{n_{combin}}s_{crit1}<s_{T}<n\cdot s_{crit1}\\ \frac{\tau_{slip}\cdot u_{f}l_{v}}{n_{combin}}s_{T}>n\cdot s_{crit1} \end{array}\right.$$

The non-linear bonding behavior in the normal direction was also represented by COMBIN39 elements. The force–displacement relationship was derived from Equation (24) as follows:

(28) $$F_{N}\left(\delta_{N}\right)=\left\{\begin{array}{c} \begin{array}{c} \frac{\frac{\tau_{max3}}{\delta_{max1}}\cdot u_{f}l_{v}\cdot \delta_{N}}{n_{combin}}0<\delta_{N}<\delta_{max1}\\ \frac{\sigma_{max1}\cdot u_{f}l_{v}-\frac{\sigma_{max1}}{n\cdot \delta_{max1}-\delta_{max1}}\cdot u_{f}l_{v}\cdot \delta_{N}}{n_{combin}}\delta_{max1}<\delta_{N}<n\cdot \delta_{max1} \end{array}\\ 0\;\delta_{N}>n\cdot \delta_{max1} \end{array}\right.$$

## 4. Adjustment of the Material Parameters of the Individual Phases

### 4.1. Filament

As formulated in Section 3.1, the elastic properties of the filaments were taken from the literature. The variables utilized in this study were derived from [21]. The variables describing the degradation behavior of the filaments, as described in Section 3.1, were also taken from the literature [38]. The variables of the material model applied to the numerical models at a later point in this study are presented in Table 2.

### 4.2. Matrix

The mineral matrix, whose material behavior was to be simulated, was used by Wilhelm [1] and is referred to as MIN-60.

The complexity of the chosen material model described in Section 3.2 required the variables to be partly estimated based on the literature and subsequently adjusted based on experimental investigations. The experimental investigations were carried out at the Institute for Construction Materials at the Technical University of Dresden. The Poisson ratio $\nu_{m}$ of the matrix was predetermined as 0.15 according to [39]. Three-point bending and compression tests were carried out to define the remaining parameters of the material model.

#### 4.2.1. Experimental Studies

The production process of the mineral matrix was adapted from the process described by Wilhelm in [1]. Following production, the mineral suspension was filled into a prism-shaped steel form, which was then sealed. Sealing reduces the risk of early drying, which, in turn, reduces the risk of cracking during hydration [39].

**Figure 7:**
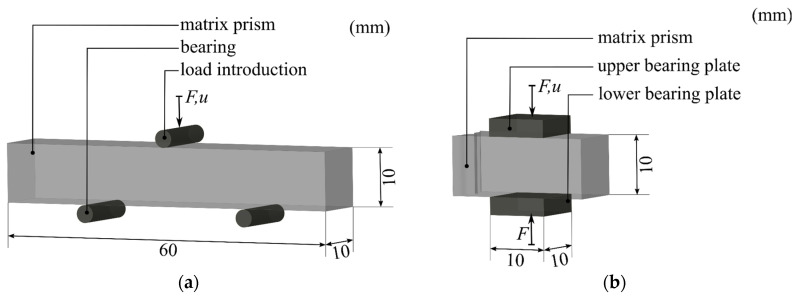
Test setup of the three-point bending (**a**) and compression tests (**b**) [42]. © Kai Zernsdorf.

The experimental tests for the adjustment of the material model of the mineral matrix, according to Section 3.2, were carried out on prisms with dimensions of 60 × 10 × 10 mm³. Sampling took place 28 days after the production of the test specimens. The tests included three-point bending and compression tests based on DIN EN 12390-3 [40] and DIN EN 12390-5 [41]. The specimen geometry and instrumentation are shown in Figure 7.

The three-point bending tests were carried out using a Zwick Roell Z1445 universal testing machine from Zwick Roell GmbH & Co. KG (Ulm, Germany) until the specimens failed. The stress was applied in a path-controlled manner at a displacement speed of 1 mm/min. The load cell used had a maximum load of 1 kN. During the testing process, the load F was recorded, as well as its associated vertical deformation u, by tracking the travel of the load roller.

Subsequently, compression tests were carried out on the undamaged prism halves. The test procedure was identical to the three-point bending tests. Only the load cell used was different, having a maximum load of 10 kN. The vertical deformation u was recorded via the displacement of the upper load introduction plate. 

#### 4.2.2. Finite Element Model and Results

**Figure 8:**
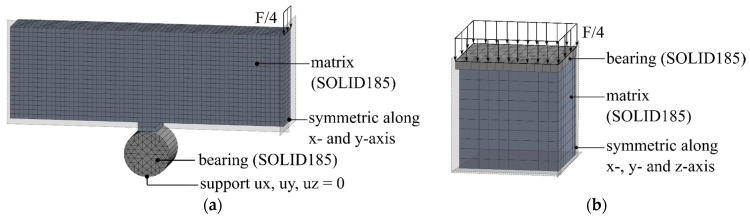
Numerical models for the bending (**a**) and compression tests (**b**). © Kai Zernsdorf.

Two models were created in the FE program Ansys for the simulation of the three-point bending and compression tests. These models are shown in Figure 8.

In the models, it was assumed that the contact between the bearing plate and the specimen, as well as the bearing or load introduction and the specimen, was frictionally engaged (Figure 8). According to the literature, the coefficient of friction was selected to be 0.57 [43]. The contact model facilitates the transmission of compressive and bond stresses. However, it is incapable of transmitting tensile stresses. For additional information regarding contact modeling with friction, see [44].

Due to existing model symmetries, the modeling of a quarter model was sufficient for both test setups. The mineral matrix, bearing, and load transfer were modeled using eight-node, three-dimensional SOLID185 elements. The elements had three translational degrees of freedom at each node. The anisotropic damage behavior of the matrix was simulated using the elastic damage microplane material model, as described in Section 3.2. The variables of the damage model, k, $\alpha^{mic}$, $\beta^{mic}$, and $\gamma_{0}^{mic}$, were varied until the experimental tests were approximated as best as possible.

**Figure 9:**
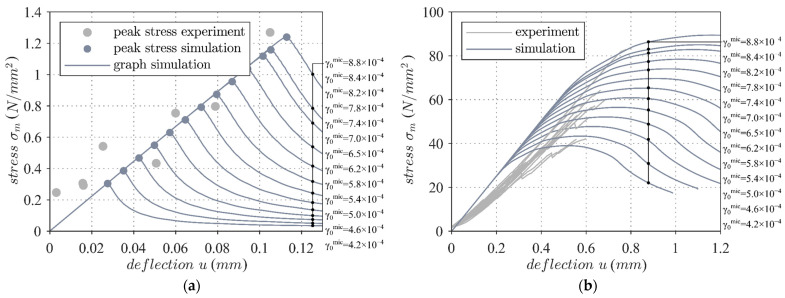
Comparison of the experimental investigation results of the mineral matrix with the numerical stress–strain graphs using a calibrated material model with varying damage threshold $\gamma_{0}^{mic}$ for the bending test (**a**) and compression test (**b**). © Kai Zernsdorf.

The material parameters of the model are listed in Table 3. The scattering material strength was considered by varying the damage threshold $\gamma_{0}$ according to the experimental test data. From Figure 9, it is clear that the force–displacement graphs of the numerical model sufficiently match the experimental results.

### 4.3. Bond between Filament and Matrix 

To adjust the variables of the bond model presented in Section 3.3, single-fiber pull-out tests were carried out at the Institute for Construction Materials at the Technical University of Dresden. The experimental tests were conducted in accordance with [45,46], as this particular test setup has proven to be efficacious in evaluating the bond behavior of a diverse range of filament–matrix combinations.

#### 4.3.1. Experimental Studies

The preparation of the mineral matrix into which the filament was incorporated was carried out according to the procedures described in Section 4.2.1. Following production, the mineral suspension was placed in a plastic mold, together with a fiber heavy tow from SGL Carbon SE (Wiesbaden, Germany) called SIGRAFIL C T50-4.4/255-E100. The bond length was 0.5 mm. The detailed manufacturing methodology of the test specimens can be found in [46]. The test specimens were removed 48 h after production and stored in an airtight container until sampling. Similar to the mineral matrix, the storage methodology should reduce the formation of cracks.

**Figure 10:**
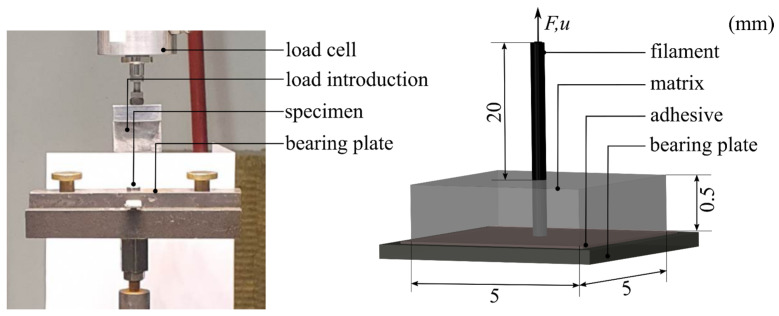
Test setup and instrumentation of the single-fiber pull-out test according to [45]. © Kai Zernsdorf.

**Figure 11:**
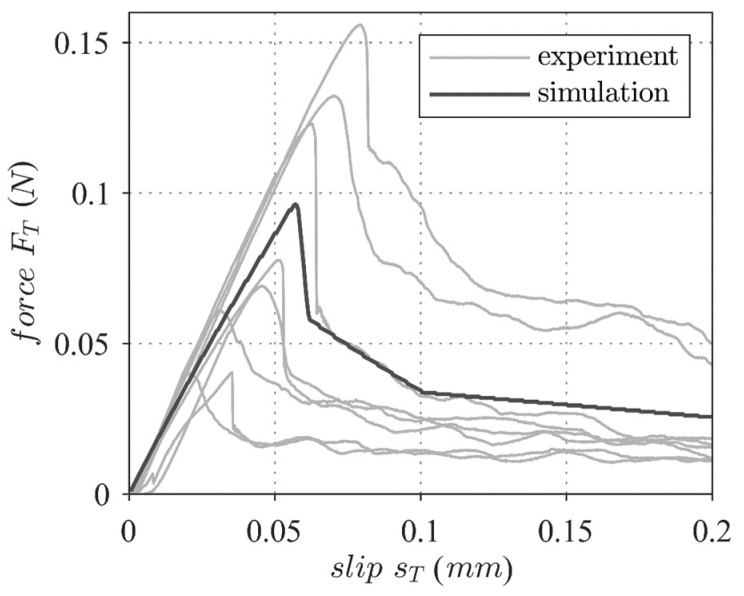
Experimental results and simulation results with the adjusted material model. © Kai Zernsdorf.

The single-fiber pull-out test was performed using a Zwick Roell Z1445 testing machine from Zwick Roell GmbH & Co. KG (Ulm, Germany) 28 days after specimen production. Before testing, the lower surface of each sample was glued to a bearing plate. The bearing plate was again anchored mechanically [46]. The free end of the filament was attached to a load application plate coupled to a load cell with a maximum capacity of 10 N [46] (Figure 10). The specimens were tested under displacement control at a constant travel speed of 0.01 mm/s [45]. During the test, the applied load and associated displacement were measured at the free end of the filament. The bond behavior was determined using eight test specimens. The experimentally determined force–extraction graphs are shown in Figure 11.

#### 4.3.2. Finite Element Model and Results

**Figure 12:**
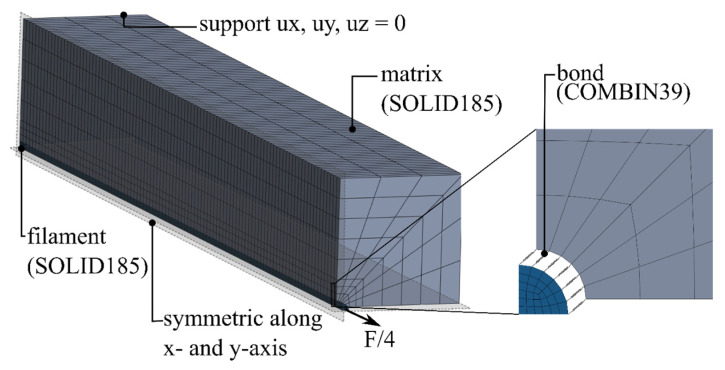
Numerical model of the single-fiber pull-out test. © Kai Zernsdorf.

The single-fiber pull-out test was simulated in the FE program Ansys in accordance with the work in [47,48,49]. Based on the model geometry, a quarter model with symmetrical boundary conditions was generated (Figure 12). The three-dimensional model had three differentiated material models. The mineral matrix was simulated using the elastic damage microplane material model. The scalar input parameters of this model are shown in Table 3. Since no filament destruction was recorded during the experimental investigations, the filaments were modeled with a linear elastic, transversely isotropic material behavior, without considering the damage, according to Table 2 (cf. Section 3.1).

The bonds between the matrix and the filaments were mapped using two-node COMBIN39 elements. These elements allowed the simulation of slip-dependent tangential and normal forces between a filament and a matrix node via the definition of a non-linear spring characteristic. The strain in the COMBIN39 elements was equivalent to the slip of the filament, where the resistance $F_{T}\left(s_{T}\right)$ acted according to Equation (27). As shown in Figure 12, seven COMBIN39 elements were arranged per element partition in the longitudinal direction of the filament.

The adjusted variables of the bond model are summarized in Table 4. The obtained simulation results exhibited the anticipated non-linear force–displacement behavior and were in good agreement with the force–displacement curves that were experimentally determined (see Figure 11).

## 5. Validation of the MCF-RVE

Using the adjusted material models of the individual phases, it was possible to carry out numerical material tests on the MCF-RVE to predict the tensile mechanical behavior of the MCF reinforcement and validate the numerical model. In the numerical tests, the side length of the MCF-RVE was $L_{RVE}=10\times 10$.

For this purpose, a deformation was induced on the edge surfaces of the MCF-RVE in the longitudinal direction of the filaments. This was equivalent to the uniaxial strain ($\varepsilon_{z}^{0}\neq 0$) and resulted in a stress response in the MCF-RVE. On the basis of this stress and strain, the stiffness behavior and degradation of the MCF-RVE could be predicted.

**Figure 13:**
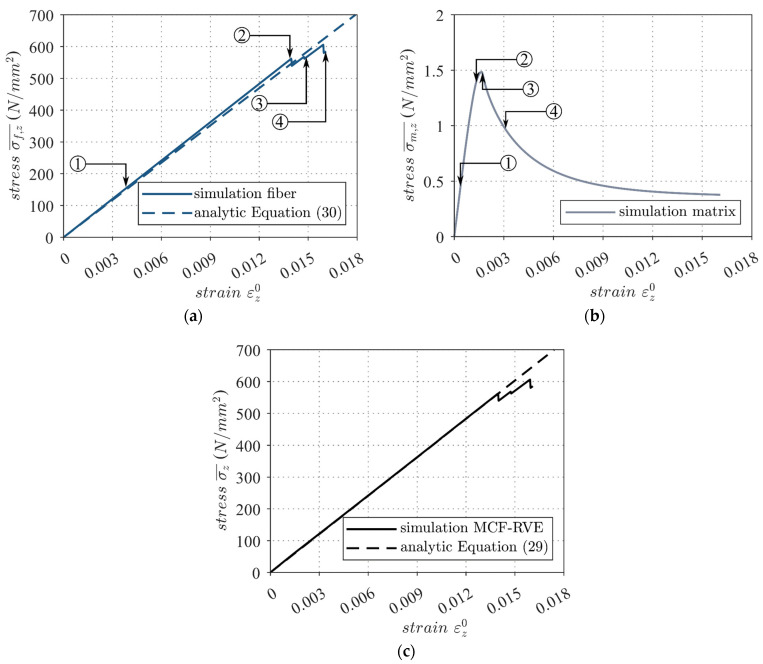
Effective stress–strain behavior of the fiber with its evolution of degradation ranges from ➀–➃, as depicted in Figure 15 (**a**), the matrix with its evolution of degradation ranges from ➀–➃, as depicted in Figure 14 (**b**), and the MCF-RVE (**c**) due to longitudinal tensile loading. © Kai Zernsdorf.

The homogenized stress $\bar{\sigma_{z}}$ of the MCF-RVE is shown in Figure 13c. Since a uniaxial strain state was imposed on the MCF-RVE, the transverse resistance of the composite led to lateral constraints, which, in turn, caused a multi-axial stress state. Consequently, in addition to the stress components in the z-direction, stresses were induced in the x- and y-directions. From the homogenized stress–strain behavior shown (Figure 13c), it is evident that the MCF-RVE initially exhibited linear elastic behavior, with an effective stiffness that was consistent with the rule of mixtures [50]:

(29) $$\bar{\sigma_{z}}\left(\varepsilon_{z}^{0}\right)=E_{f,z}\cdot \varphi \cdot \varepsilon_{z}^{0}+E_{m}\cdot \left(1-\varphi \right)\cdot \varepsilon_{z}^{0}$$

**Figure 14:**
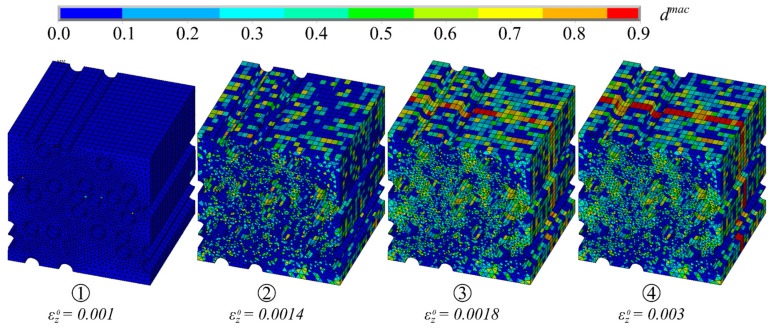
Evolution of matrix degradation $d^{mac}$. © Kai Zernsdorf.

The developing microscopic damage in the matrix is shown in Figure 14. It became apparent that the damage initially formed randomly throughout the MCF-RVE. With increasing load, local damage zones developed in the matrix, which led to a local and, later, to a global softening behavior at a strain of $\varepsilon_{z}^{0}=0.0018$ (Figure 13b and Figure 14). The damage occurred orthogonal to the direction of stress. Damage to the matrix elements, in turn, caused stress concentrations in adjacent filament elements, increasing the probability of failure of the filament elements at these locations. The progressing damage, as well as the subsequent exponential reduction in the stiffness of the mineral matrix within the damage band (Figure 13b), resulted in a stress concentration within the filaments. Thus, at this point, the macroscopic stiffness of the composite approximated the stiffness of the filaments. Analytically, this can be expressed with the following function:

(30) $$\bar{\sigma_{f,z}}\left(\varepsilon_{z}^{0}\right)=E_{f,z}\cdot \varphi \cdot \varepsilon_{z}^{0}$$

This evaluation shows that the MCF-RVE had a similar stiffness behavior to conventional fiber-reinforced polymers. Since the stiffness of the filaments $E_{f,z}$ was significantly greater than the stiffness of the matrix $E_{m}$, the following approximation could be applied until the first filament damage (cf. Figure 13a,c):

(31) $$E_{f,z}\cdot \varphi \cdot \varepsilon_{z}^{0}+E_{m}\cdot \left(1-\varphi \right)\cdot \varepsilon_{z}^{0}≅E_{f,z}\cdot \varphi \cdot \varepsilon_{z}^{0}$$

**Figure 15:**
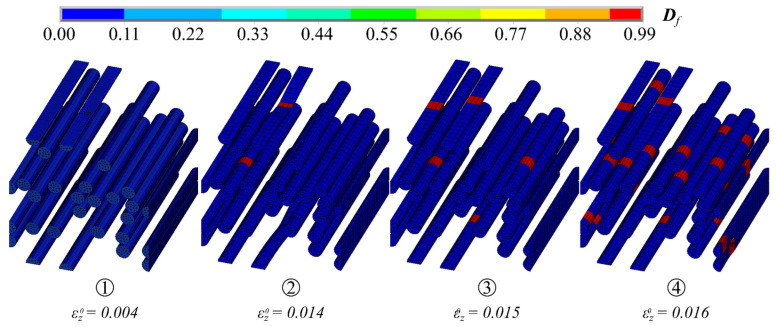
Evolution of filament degradation $D_{f}$. © Kai Zernsdorf.

Increasing the strain led to the successive degradation of individual filament elements in the MCF-RVE, which, in turn, led to stress concentrations on adjacent elements until the filaments were destroyed. This resulted in a non-linear stress–strain response commencing at a strain of approximately $\varepsilon_{z}^{0}=0.014$ (Figure 13a and Figure 15). The first filament breakage was followed by the failure of further filaments (Figure 15). The breaking point of a filament depended on defects within the filament. Based on the analysis conducted, it appeared that the filaments did not fail in a uniform plane. This was also observed in [12] for fiber-reinforced polymers.

In conclusion, the filaments served to absorb tensile stresses in the MCF-RVE, and the composite matrix exhibited early brittle cracking, which affected the stiffness of the MCF-RVE (cf. Figure 13b and Figure 14). After cracking, crack-bridging effects occurred, which were considered in the bond model. These effects, together with the gradual destruction of the filaments (cf. Figure 13a and Figure 15), influenced the tensile-load-bearing behavior of the developed model depicted in Figure 13c.

**Figure 16:**
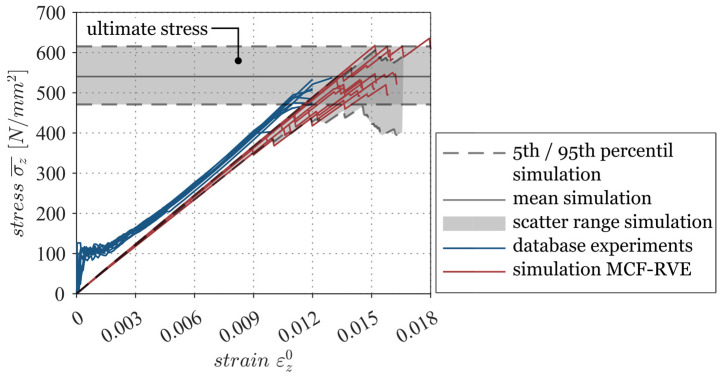
Comparison between the numerical results of the MCF-RVE and the experimental results reported by Wilhelm [1]. © Kai Zernsdorf.

**Figure 17:**
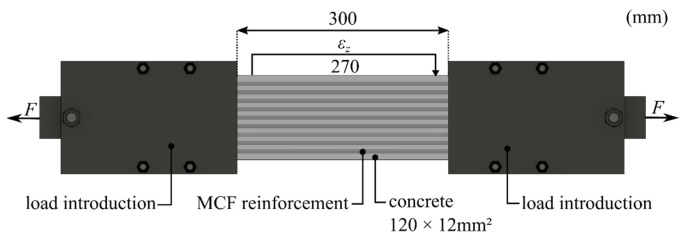
Test setup of a uniaxial tension test according to Wilhelm [1]. © Kai Zernsdorf.

Subsequently, validation of the MCF-RVE was carried out. For this purpose, ten randomized numerical models (Figure S5) were generated, and the numerical results of these models were compared with the experimental investigations conducted by Wilhelm [1] (Figure 16). Cross-sections of the numerical models are depicted in Figure S5. Wilhelm [1] carried out uniaxial tensile tests on MCF-reinforced concrete specimens. The geometry of the test specimens, as well as the test setup, is shown in Figure 17.

It should be noted that the test results of the constructed MCF-RVE did not replicate the results of the uniaxial tension test of the reinforced concrete slab depicted in Figure 17. Only the stiffness and strength of the reinforcement were represented in the MCF-RVE. Moreover, the boundary effects of the experiment, such as the anchorage systems of the test specimens and the resulting transverse pressure on the reinforcement, were not considered, nor was the influence of the surrounding concrete on the reinforcement. In addition, the length of the representative volume element was significantly smaller than the length in the experimental trial.

From Figure 16, it is evident that the homogenized stress–strain behavior of the MCF-RVE correlated with the experimental investigations. In particular, congruence was achieved regarding the stiffness of the reinforcement. Successive filament degradation was evident in the experimental studies carried out by Wilhelm [1], as well as in the numerical results of the MCF-RVE. Commencing at a strain of $\varepsilon_{z}^{0}\approx 0.011$, the experimental graphs show a flattening, which could be attributed to the damage of individual filaments in the reinforcement. In the numerical investigations, this damage occurred from a strain of $\varepsilon_{z}^{0}\approx 0.01$. In the experimental investigations, the maximum stress that could be absorbed was approximately 510 N/mm² at a strain of $\varepsilon_{z}^{0}\approx 0.012$. In the MCF-RVE, on the other hand, the mean ultimate load-bearing capacity was about 540 N/mm² at a mean ultimate strain of $\varepsilon_{z}^{0}\approx 0.015$. The ultimate stresses that were experimentally determined were approximately within the numerical failure range as defined by the failure limit lines for the upper (95th percentile) and lower (5th percentile) ranges. Since the scalar damage variables $\sigma_{0}$ and m were taken from the literature and not determined experimentally, this small deviation from the maximum tensile load capacity is considered acceptable.

Finally, we concluded that the constructed MCF-RVE captured the essential mechanical properties of MCF reinforcement and could, therefore, be considered representative for further investigation. The numerical results of the MCF-RVE under tensile stress were comparable to the uniaxial tensile tests carried out on MCF-reinforced concrete slabs in a cracked state. Therefore, the MCF-RVE provided valid results, making it possible to predict the tensile mechanical properties of the MCF reinforcement.

## 6. Sensitivity Analysis

Section 5 demonstrated that the parameters of the filament, in particular, exert a significant impact on the stress–strain behavior of the MCF-RVE. Consequently, in this section, the sensitivity of the numerical solution regarding the input parameters of the material properties—such as the modulus of elasticity of the filament, filament strength, and Weibull module—is investigated. The stress–strain behavior was used as a criterion for evaluating the sensitivity of the simulation to the input parameters.

### 6.1. Sensitivity of Modulus of Elasticity E_f,z_

**Figure 18:**
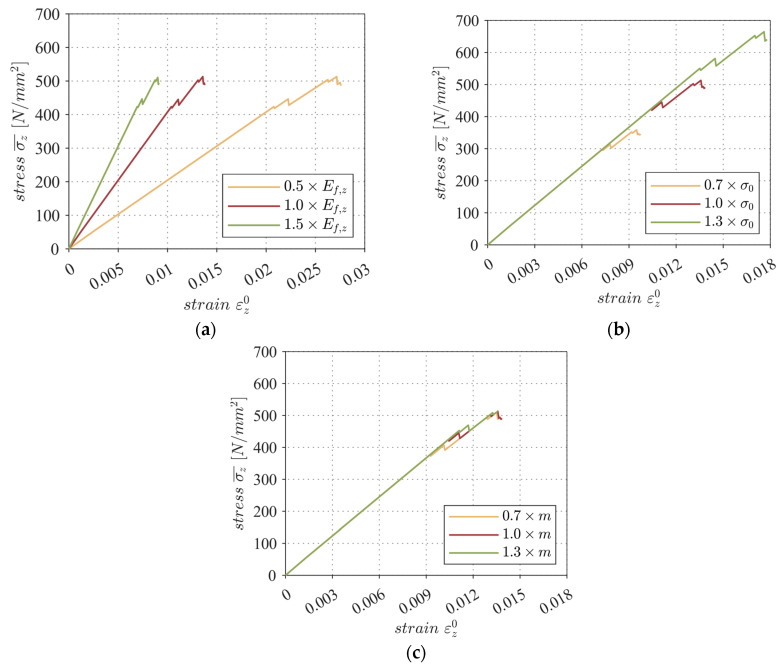
Sensitivity to modulus of elasticity (**a**), tensile strength (**b**), and Weibull module (**c**) due to longitudinal tensile loading. © Kai Zernsdorf.

The modulus of elasticity was changed from $0.5\times E_{f,z}$ to $1.5\times E_{f,z}$, where $E_{f,z}$ is the modulus of elasticity taken from Table 2. The numerical outcomes depicted in Figure 18a were generated utilizing the geometric model depicted in Figure S5a. The stiffness of the MCF-RVE increased in direct proportion to the increasing filament stiffness. The failure load was not influenced by the change in the modulus of elasticity of the filament.

### 6.2. Sensitivity of Tensile Strength $\sigma_{0}$

The filament strength $\sigma_{0}$ taken from Table 2 was changed from $0.7\times \sigma_{0}$ to $1.3\times \sigma_{0}$. The results presented in Figure 18b were generated using the geometric model shown in Figure S5a. As expected, the ultimate stress of the MCF-RVE increased proportionally to the increasing tensile strength of the filament. It is important to note that neither the stiffness nor the progressive filament degradation were affected by the adjustment of the parameter $\sigma_{0}$.

### 6.3. Sensitivity of Weibull Module m

The Weibull module m taken from Table 2 was changed from 0.7×m to 1.3×m. Figure 18c illustrates that the probability of filament failure was reduced as the Weibull module increased. In the stress–strain graph, this is indicated by the progressively higher stress levels at which the first filament rupture occurs. Nonetheless, the ultimate stress within the MCF-RVE remained constant, as did the increase in all graphs.

## 7. Conclusions

This paper presented an approach for the numerical characterization of mineral-impregnated carbon fiber reinforcement (MCF reinforcement) according to [1] using representative volume elements (RVEs). In the first step, a suitable geometric model, including the corresponding boundary conditions, was generated, which contained the individual components of the MCF reinforcement. Subsequently, constitutive regularities of the individual material phases of the filaments, matrix, and bonds were defined, and their implementation in the numerical model was performed. In the model, the filaments exhibited transversely isotropic material properties, and we applied a statistical, one-dimensional damage model, which enabled successive filament destruction. The material model of the mineral matrix depicted the multidimensional damage behavior of the material. The bonds between the filaments and matrix were defined using a non-linear spring model. The following are the findings of this study:

1. The MCF-RVE constructed using the adjusted material models enabled the simulation of the degradation behavior as well as the prediction of the effective mechanical behavior of the MCF reinforcement in the longitudinal filament direction. The numerical results were compared with the experimental results. The constructed MCF-RVE was able to capture the dominant mechanical properties of the MCF reinforcement for the considered load type.
2. The robustness of the numerical model was evaluated by a sensitivity analysis. The model exhibits high stability and is capable of handling variations in model parameters. The reliability of Wilhelm’s validation set [1] can be attributed to the quality of its data. Nonetheless, it should be noted that the calibration data for certain parameters were obtained from specialized literature rather than being determined through experimental testing.
3. The definition of the elastic and damage mechanical parameters of the filaments based on the literature provided adequate results. An experimental determination of the parameters was not necessary, but an adjustment of the average filament strength $\sigma_{0}$ and the Weibull module m could lead to more exact results. The parameters $\sigma_{0}$, m, and $E_{f,3}$ had a decisive influence on the model results.
4. The validation of the MCF-RVE showed that the influence of the mineral phase on the stiffness and degradation behavior was not significant, as the stiffness of the mineral matrix was significantly smaller than that of the filaments. However, it was necessary to quantify the elastic and tensile mechanical properties of the mineral matrix in advance, as increasing stiffness and strength were accompanied by an increase in the influence of the matrix on the homogenized stress–strain response of the MCF-RVE. In the simulation, the ultimate strength of the MCF reinforcement was approximately 600 N/mm² at a strain of 0.016.
5. The analytical bond model of Brameshuber and Banholzer [27] and the spring model of Ngo and Scrodelis [26] enabled the simulation of the bond behavior between the filaments and the mineral matrix. The spring model could be used in the simulation of single-fiber pull-out tests, as well as within the MCF-RVE, for bond simulations. A non-linear simulation of the bond between the reinforcement and the matrix showed no significant influence on the stress–strain response of the MCF-RVE, but a correct formulation of the bond was necessary to quantify the damage mechanisms in the matrix, as well as in the filaments.

The presented approach provides a more profound understanding of MCF reinforcement and valuable insights into its microstructure. It is possible to derive the mechanical properties of MCF reinforcement from the model. The results of this study enable the development of a one-dimensional material law for MCF reinforcement rebars. By utilizing this material law, it will be feasible to simulate structures that are reinforced with mineral-impregnated carbon fibers. In addition, geometric and material-specific parameters could be used to further optimize the material. Approaches to this are described in [12].

## Figures and Tables

**Table 1:** Sequence of macros for filament degradation [22].

1.	Read the strain of the element at time *t*
	$\varepsilon_{f,t}$
2.	Calculate the stress at time *t*
	$\sigma_{f,t}$
3.	Update the history variable
	${\hat{\gamma }}_{t}=max\left({\hat{\gamma }}_{t-1},\sigma_{f,t}\right)$
4.	Calculate the degradation
	$D_{f}\left({\hat{\gamma }}_{t}\right)=\left\{\begin{array}{c} 0\;für\;{\hat{\gamma }}_{t}<X_{f,z}\\ 1\;für\;{\hat{\gamma }}_{t}\geq X_{f,z} \end{array}\right.$
5.	Determine the stress and the stiffness tensor at time *t*
	$\sigma_{f}=C_{f}\left(1-D_{f}\left({\hat{\gamma }}_{t}\right)\right):\varepsilon_{f}$ $C_{f}^{D}=C_{f}\left(1-D_{f}\left({\hat{\gamma }}_{t}\right)\right)$

**Table 2:** Model parameters for a carbon filament [1,21,38].

Material Property	Value	Source
$\mathrm{Filament}\;\mathrm{diameter},\;d_{f}$ (mm)	0.0069	[1]
Modulus of elasticity		
$E_{f,x}=E_{f,y}$ (N/mm²)	15,000	[21]
$E_{f,z}$ (N/mm²)	230,000	[21]
$\mathrm{Transverse}\;\mathrm{Poisson}’s\;\mathrm{ratio},\;\upsilon_{f,zy}$	0.2	[21]
Shear modulus		
$G_{f,xy}$ (N/mm²)	15,000	[21]
$G_{f,yz}$ (N/mm²)	7000	[21]
Weibull parameters		
$\sigma_{0}$ (N/mm²)	3170	[38]
m	5.1	[38]
$L_{0}$ (mm)	25	[38]

**Table 3:** Identified model parameters of the mineral matrix (average values; standard deviations in parenthesis).

Elasticity Variables		Damage Model			
$E_{m}$ (N/mm²)	$\nu_{m}$	k	$\alpha^{mic}$	$\beta^{mic}$	$\gamma_{0}^{mic}$
1350	0.15	180	0.95	40	0.00065 (0.000163)

**Table 4:** Identified parameters of the bond model.

$\tau_{max1}$ (N/mm²)	$\tau_{max2}$ (N/mm²)	$\tau_{slip}$ (N/mm²)	$s_{crit1}$ (mm)	n
7.85	4.71	3.00	0.055	1.82

## Data Availability

Data are contained within the article.

## Supplementary Materials

The following supporting information can be downloaded at https://www.mdpi.com/article/10.3390/ma17030737/s1. Figure S1: Exemplary microstructures with a fiber volume fraction of 17% in a periodic topology with dimensions of $L_{RVE}=3\times 3$ (a), $L_{RVE}=5\times 5$ (b), $L_{RVE}=8\times 8$ (c), $L_{RVE}=10\times 10$ (d), and $L_{RVE}=12\times 12$ (e); Figure S2: Effective stress–strain behavior of the MCF-RVE with different side lengths (a) and simulation time vs. side length of the MCF-RVE (b); Figure S3: Flowchart of randomly distributed filaments using MATLAB; Figure S4: Supplementary Figures of the flowchart for visualization if the filament does not penetrate the RVE edge (a), if the filament penetrates the RVE edge (b), if the filament overlaps (c), and if the filament does not overlap (d); Figure S5: Randomly generated model geometries one (a), two (b), three (c), four (d), five (e), six (f), seven (g), eight (h), nine (i), and ten (j) according to Section 2 with dimensions of $L_{RVE}=10\times 10$.

## Abbreviations

**Acronyms**	
ESEM	Environmental scanning electron microscope
FE/FEM	Finite element method
MCF	Mineral-impregnated carbon fiber
RVE	Representative volume element
**Latin letters**	
C	Stiffness tensor
$\bar{C}$	Homogenized stiffness tensor
$C_{f}^{D}$	Damaged stiffness tensor of the filament
$D_{f}$	Damage tensor of the filament
d	Variable of damage
dev	Deviatoric part of the second-order unit tensor
$d_{f}$	Filament diameter
E	Modulus of elasticity
F	Force
$F_{N}$	Force with direction normal to the filament
$F_{T}$	Force with direction tangential to the filament
G	Shear modulus
$i,\;i^{+},\;i^{-}$	Node pair i $\;\mathrm{with}\;\mathrm{node}\;i^{+}$ $\;\mathrm{and}\;\mathrm{node}\;i^{-}$
K	Bulk modulus
k	Ratio of pressure to tensile strength
$k_{0}$ $,\;k_{1}$ $,\;k_{2}$	Parameters defining the shape of the damage surface
L	Length of an element
$L_{0}$	Gauge length
$L_{RVE}$	Side length of the representative volume element
$l_{v}$	Bond length
m	Weibull module
n	Factor defining the slip at the transition to the sliding shear stress
$s_{crit1}$	$\mathrm{Slip}\;\mathrm{at}\;\mathrm{shear}\;\mathrm{stress}\;\tau_{max1}$
$s_{T}$	Slip
t	Time step within the simulation
u	Displacement
$u^{*}$	Fluctuation within the displacement field
$u_{f}$	Filament circumference
V	Volume of the representative volume element
vol	Volumetric part of the second-order unit tensor
x≙1	1. Spatial direction orthogonal to the filament
y≙2	2. Spatial direction orthogonal to the filament
z≙3	Spatial direction parallel to the filament
${\left(∎\right)}_{f},\;{\left(∎\right)}_{m},\;{\left(∎\right)}_{int}$	Indexing for filament, matrix, and bond between filament and matrix
${\left(∎\right)}^{mac}$	Indexing for quantities on the microplane integrated over the solid angle of a unit sphere with a surface area of 4π over the angle Ω
${\left(∎\right)}^{mic}$	Parameter on the microplane
**Greek letters**	
$\alpha^{mic}$	Maximum degree of degradation
$\beta^{mic}$	Degradation rate
$\gamma_{0}^{mic}$	Damage threshold
${\hat{\gamma }}_{t}$	History parameter
Δx	Distance between opposite node pairs
$\delta_{max1}$	$\mathrm{Relative}\;\mathrm{displacement}\;\mathrm{upon}\;\sigma_{max1}$
$\delta_{N}$	Relative displacement between a filament and the matrix perpendicular to the filament
ε	Strain
$\varepsilon_{0}$	Linear displacement component within a displacement field
$\eta^{mic}$	Equivalent strain energy
ν	Poisson’s ratio
σ	Stress
$\bar{\sigma }$	Homogenized stress of the representative volume element
$\sigma_{0}$	Characteristic tensile strength of the filament
$\sigma^{loc}$	Stress of an element within the representative volume element
$\sigma_{max1}$	Maximum normal stress between filament and matrix
$\sigma_{N}$	Effective normal stress between filament and matrix
$\tau_{max1}$	Maximum shear stress between filament and matrix
$\tau_{max2}$	$\mathrm{Shear}\;\mathrm{stress}\;\mathrm{after}\;\mathrm{reaching}\;\tau_{max1}$
$\tau_{slip}$	Slip shear stress
$\tau_{T}$	Shear stress
φ	Fiber volume fraction
$X_{z}$	Random number between 0 and 1
$X_{f,z}$	Tensile strength of a filament element
ψ	Energy input
Ω	Angle of a unit sphere with an area of 4π
