# Supplementary material for: Numerical Material Testing of Mineral-Impregnated Carbon Fiber Reinforcement for Concrete
Source: Materials (Basel). 2024 Feb 3;17(3):737. doi: 10.3390/ma17030737 (PMC10856107; doi:10.3390/ma17030737)
Supplement: Supplementary file 1 [file materials-17-00737-s001.zip › materials-2805822-supplementary.pdf]

Supplementary materials

Numerical Material Testing of Mineral-Impregnated Carbon Fiber Reinforcement for Concrete

Kai Zernsdorf [1]*, Viktor Mechtcherine [2], Manfred Curbach [3] and Thomas Bösche [1]

[1] Department of Structural Engineering, Hochschule für Technik und Wirtschaft Dresden, 01069 Dresden, Germany; thomas.boesche@htw-dresden.de
[2] Institute of Construction Materials, Technische Universität Dresden, 01069 Dresden, Germany; viktor.mechtcherine@tu-dresden.de
[3] Institute of Concrete Structures, Technische Universität Dresden, 01069 Dresden, Germany; manfred.curbach@tu-dresden.de
* Correspondence: kai.zernsdorf@htw-dresden.de; Tel.: +49-351-462-2082

1. Sensitivity Analysis of the Side Length L_{RVE}

One of the most important issues encountered in the simulations of the MCF-RVE was the choice of the parameter L_{RVE}. As discussed in Section 2, the model must be sufficiently large to allow for enough random heterogeneities. In this particular instance, it is feasible to simulate the successive degradation of the filaments within the MCF-RVE. Since there were no estimates of the parameter available in the literature, a parametric study was performed to determine the optimal value of the side length. Figure S1 illustrates the investigated models with different side lengths. Figure S2a shows the stress–strain graphs. The successive degradation of filaments and the resulting non-linear stress–strain behavior can only be represented by a model size of $L_{RVE} = 10 \times 10$ or larger.

For reference, the finite element simulations were performed on a Microsoft Windows system with 16 CPUs. The numerical simulation's time t is subject to significant variation, primarily attributed to the chosen model size. As depicted in Figure S2b, these fluctuations can span from 1.5 to 12 hours.

Finally, we concluded that the model size of $L_{RVE} = 10 \times 10$ is sufficient to represent a successive degradation within the numerical simulation.

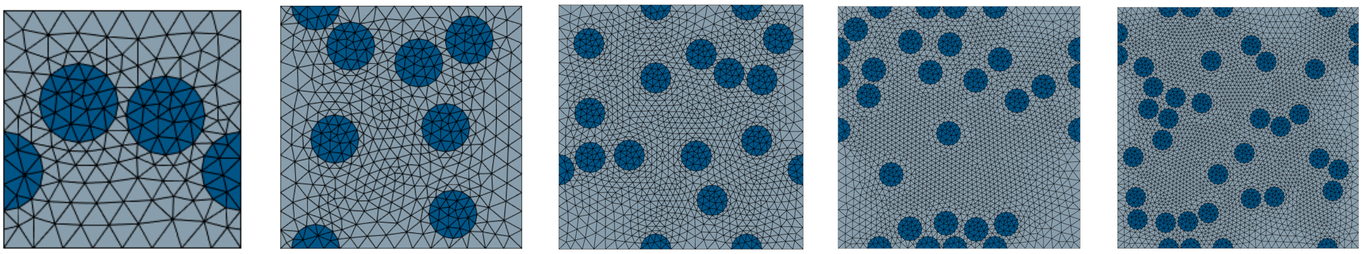

(**a**) (**b**) (**c**) (**d**) (**e**)

Figure S1. Exemplary microstructures with a fiber volume fraction of 17% in a periodic topology with dimensions of $L_{RVE} = 3 \times 3$ (**a**), $L_{RVE} = 5 \times 5$ (**b**), $L_{RVE} = 8 \times 8$ (**c**), $L_{RVE} = 10 \times 10$ (**d**) and $L_{RVE} = 12 \times 12$ (**e**). © Kai Zernsdorf.

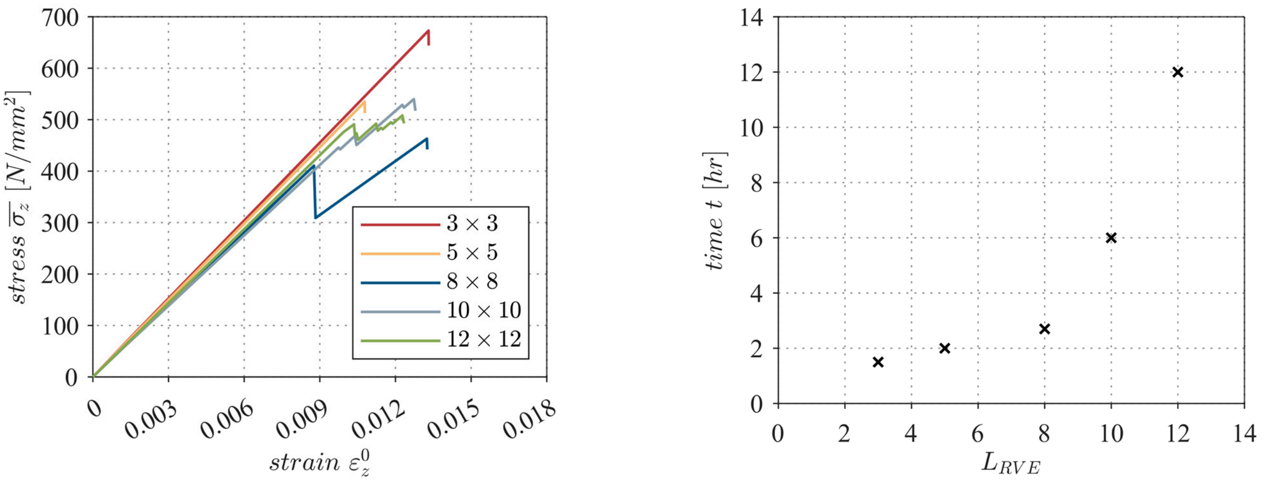

(**a**) (**b**)

Figure S2. Effective stress–strain behavior of the MCF-RVE with different side lengths (**a**) and simulation time vs. side length of the MCF-RVE (**b**) . © Kai Zernsdorf.

2. Flowchart of Randomly Distributed Filaments

A randomized positioning algorithm for filaments within a representative volume element was implemented using the program MATLAB (version 9.12). Figure S3 provides a flowchart of the programmed routine. The individual steps of the algorithm are outlined in Figure S4a-d.

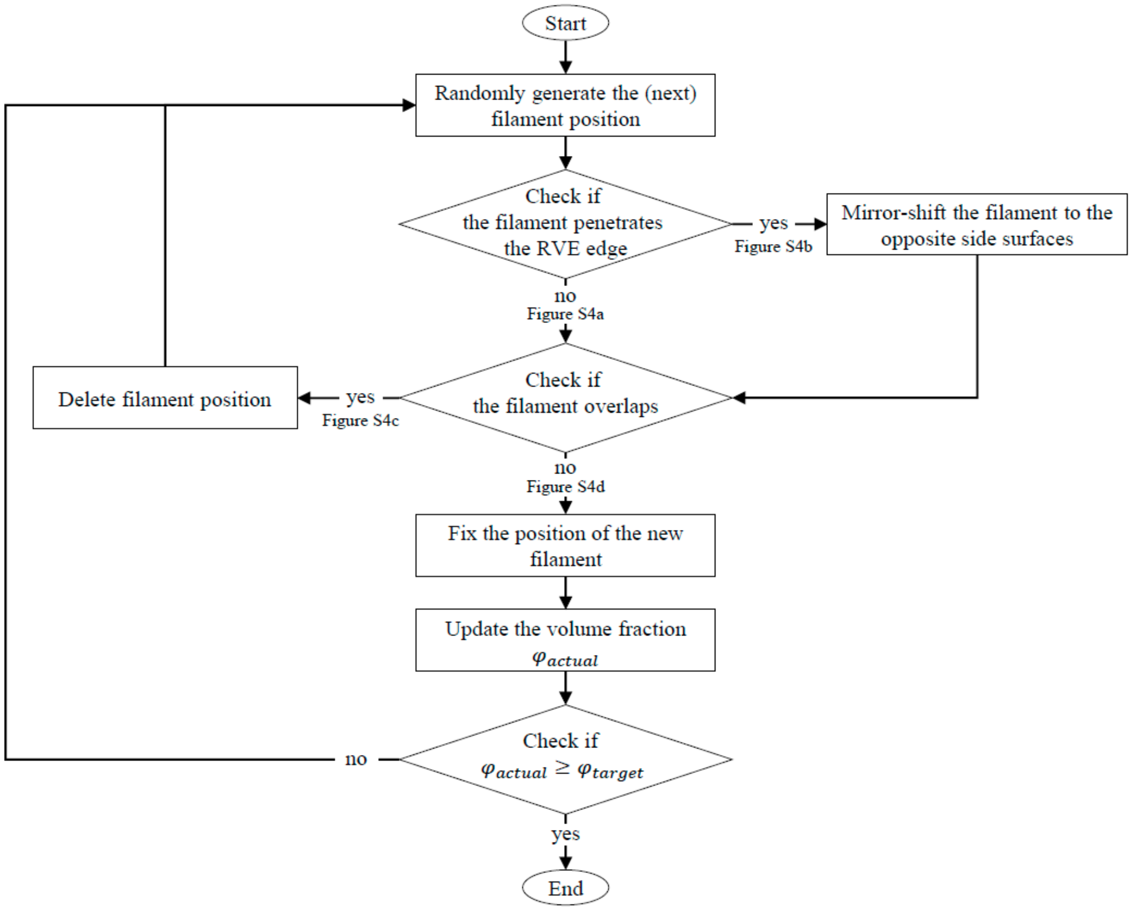

Figure S3. Flowchart of randomly distributed filaments using MATLAB. © Kai Zernsdorf.

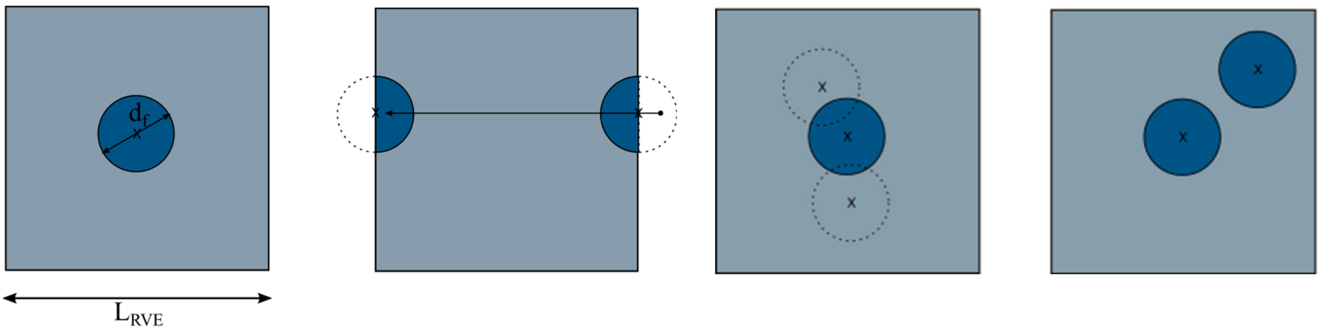

(**a**) (**b**) (**c**) (**d**)

Figure S4. Supplementary Figures of the flowchart for visualization if the filament does not penetrate the RVE edge (**a**), if the filament penetrates the RVE edge (**b**), if the filament overlaps (**c**), and if the filament does not overlap (**d**). © Kai Zernsdorf.

3. Randomly Generated Model Geometries

Figure S5a–j illustrate the cross-sections of the representative volume elements that were generated using the algorithm described in Section 2. In Section 5, Figure 16, the model outcomes were compared with experimental investigations.

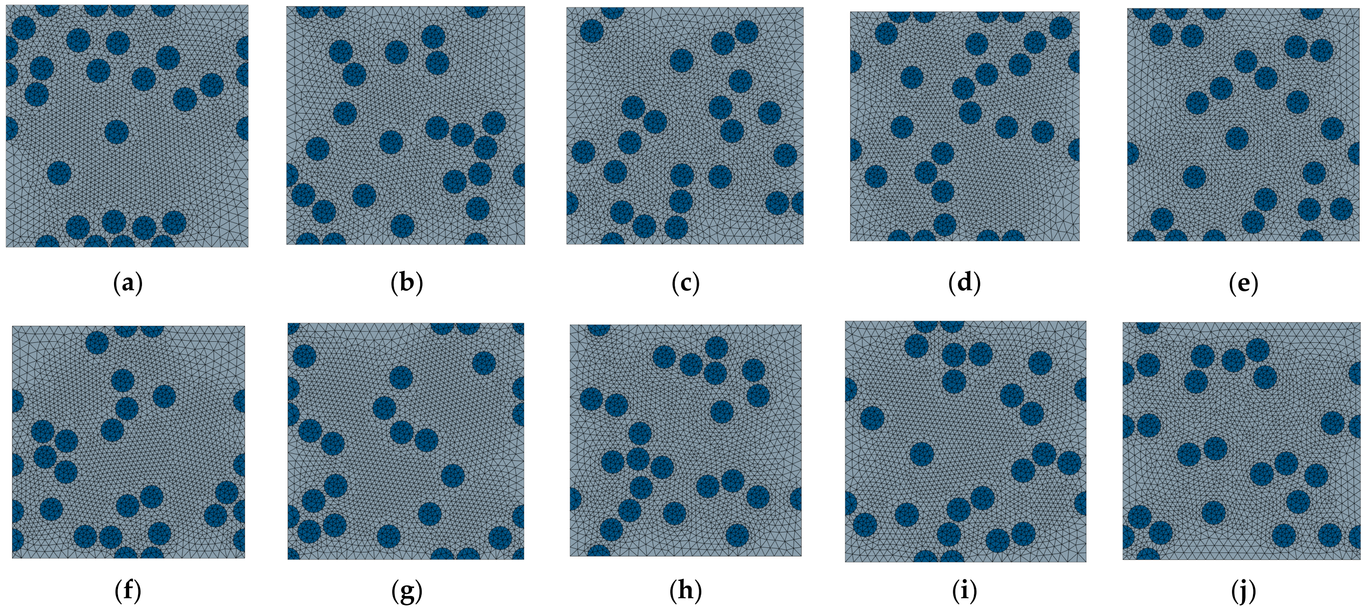

Figure S5. Randomly generated model geometries one (**a**), two (**b**), three (**c**), four (**d**), five (**e**), six (**f**), seven (**g**), eight (**h**), nine (**i**), and ten (**j**) according to Section 2 with dimensions of L_{RVE} = 10 × 10. © Kai Zernsdorf.
